# MARCH1 encourages tumour progression of hepatocellular carcinoma via regulation of PI3K‐AKT‐β‐catenin pathways

**DOI:** 10.1111/jcmm.14235

**Published:** 2019-02-22

**Authors:** Lulu Xie, Hanhan Dai, Minjing Li, Wei Yang, Guohua Yu, Xia Wang, Peiyuan Wang, Wei Liu, Xuemei Hu, Mingdong Zhao

**Affiliations:** ^1^ Department of Imaging Binzhou Medical University Yantai China; ^2^ Medicine and Pharmacy Research Center Binzhou Medical University Yantai China; ^3^ Department of Pathology Yu Huang Ding Hospital Yantai China; ^4^ Department of Oral Pathology Binzhou Medical University Yantai China; ^5^ Department of Immunology Binzhou Medical University Yantai China

**Keywords:** apoptosis, hepatocellular carcinoma, Invasion, MARCH1, migration, nude mouse model, pathways, pirarubicin, proliferation

## Abstract

Membrane‐associated RING‐CH‐1 (MARCH1) is a membrane‐anchored E3 ubiquitin ligase that is involved in a variety of cellular processes. MARCH1 was aberrantly expressed as a tumour promoter in ovarian cancer, but the signalling about the molecular mechanism has not yet been fully illuminated. Here, we first determined that MARCH1 was obviously highly expressed in human hepatocellular carcinoma samples and cells. In addition, our findings demonstrated that the proliferation, migration and invasion of hepatocellular carcinoma were suppressed, but the apoptosis was increased, as a result of MARCH1 knockdown by either siRNA targeting MARCH1 or pirarubicin treatment. Conversely, the proliferation, migration and invasion of hepatocellular carcinoma were obviously accelerated, and the apoptosis was decreased, by transfecting the MARCH1 plasmid to make MARCH1 overexpressed. Moreover, in vivo, the results exhibited a significant inhibition of the growth of hepatocellular carcinoma in nude mice, which were given an intra‐tumour injection of siRNA targeting MARCH1. Furthermore, our study concluded that MARCH1 functions as a tumour promoter, and its role was up‐regulated the PI3K‐AKT‐β‐catenin pathways both in vitro and in vivo. In summary, our work determined that MARCH1 has an important role in the development and progression of hepatocellular carcinoma and may be used as a novel potential molecular therapeutic target in the future treatment of hepatocellular carcinoma.

## 1 INTRODUCTION

Liver cancer is a significant health problem, with 782 500 new cases and 745 500 deaths annually across the world. Moreover, about 50% of the total number of liver cancer cases and deaths worldwide occur in China.^1^ Liver cancer is the second leading cause of cancer‐related deaths globally. Approximately 90% of primary liver cancers are hepatocellular carcinoma (HCC).^1^, ^2^ HCC usually appears in patients with cirrhosis related to various etiologies. The current effective therapies for the treatment of different stages of HCC include hepatic resection, liver transplantation, tumour ablation, chemoembolization and systemic therapy according to the Barcelona Clinic Liver Cancer staging system.^3^ In recent years, combination therapy with pirarubicin (THP) in transarterial chemoembolization (TACE) and Sorafenib has been identified as being potentially useful as a first‐line treatment for advanced HCC patients.^4^ However, most patients with HCC are diagnosed at late stages, when the beneficial treatments of hepatic resection, ablation and liver transplantation cannot be applied; thus, only a minority of patients with early‐stage HCC are eligible for this procedure.^5^, ^6^ It is well known that hepatocarcinogenesis is a complex multi‐step process, that the altering of many signalling cascades could affect important oncogenes and tumour suppressors, and that molecular‐targeted therapies, such as Sorafenib, which is a small molecular protein kinase inhibitor, could be effective in treating advanced cancer.^3^, ^7^ Therefore, it is urgent to explore more efficient molecular‐targeted therapeutic strategies for advanced HCC treatment.

Membrane‐associated RING‐CH‐1 (MARCH1) is a member of the membrane‐associated RING‐CH (MARCH) family of E3 ubiquitin ligase and a negative regulator of adaptive immunity.^8^ Ubiquitination was recently identified as an important and specific process, participating in several protein degradation and cell signalling pathways through the close cooperation of three enzymes: the ubiquitin‐activating enzyme E1, the ubiquitin‐conjugating enzyme E2 and the ubiquitin‐ligase enzyme E3.^9^, ^10^ MARCH1 is primarily expressed in antigen‐presenting cells and mediates the ubiquitination of MHCII and CD86 in dendritic cells (DCs) to control DC‐mediated Treg cells.^11^, ^12^ Previous studies have focused on the role of MARCH1 in the immune system. Recently, a study demonstrated that MARCH1 was overexpressed in ovarian cancer tissues, silencing MARCH1 inhibits the proliferation, migration and invasion of the ovarian cancer cells by down‐regulating the NF‐κB and Wnt/β‐catenin pathways.^13^ These data suggest that MARCH1 may be a proto‐oncogene that promotes tumour progression and, hence, a potential molecular target for cancer therapy.

In this study, our data demonstrate that MARCH1 is highly expressed in HCC, and a high level of MARCH1 has a powerful functional effect on HCC. Additionally, further investigation reveals that the induction of the proliferation, migration, invasion and apoptosis of HCC by MARCH1 was mediated through the PI3K‐AKT‐β‐catenin signalling pathway in vitro and in vivo. This finding suggests that MARCH1 is a tumour promoter in hepatocellular carcinoma and that targeting MARCH1 may be an effective HCC therapy.

## 2 MATERIALS AND METHODS

### 2.1 HCC clinical samples

For the tissue samples, 14 clinical HCC samples and distal normal tissues were collected from patients who had undergone HCC resection at the Yu Huang Ding Hospital. The patients who were recruited for this study had not received chemotherapy or radiotherapy before surgery, and written informed consent was received from all the participants. This study was approved by the Ethics Committee of the Yu Huang Ding Hospital.

### 2.2 Cell lines and cell culture

Human HCC cell lines (HepG2 and Hep3B) and normal human liver cell lines (HL‐7702 and HHL‐5) were obtained from the Cell Research Institute of the Chinese Academy of Sciences (Shanghai, China). The HCC cell lines were cultured in DMEM with high glucose (Hyclone, Logan, UT, USA), and the normal liver cell lines were cultured in RPMI medium modified (Hyclone, USA), and supplemented with 10% foetal bovine serum (FBS; gibco, Waltham, MA, USA) and 100 U/mL penicillin, as well as 100 μg/mL streptomycin (Solarbio, Beijing, China). All the cells were incubated at 37°C in a humidified atmosphere containing 5% CO_2_.

### 2.3 Antibodies and reagents

Membrane‐associated RING‐CH‐1 (Bioss, bs‐9335, Beijing, China), Phospho‐AKT Ser473 (SAB, 11054, Randallstown, MD, USA), total AKT (SAB, 21054), GAPDH (Proteintech, 10494‐1‐AP, Wuhan, Hubei, China), PI3K p110 β (Proteintech, 20584‐1‐AP), β‐catenin (Proteintech, 51067‐2‐AP), Mcl‐1 (Proteintech, 16225‐1‐AP), Bcl‐2 (Proteintech, 12789‐1‐AP), Cleaved caspase‐3 (CST, 9661, Fall River, MA, USA), Cleaved caspase‐7 (CST, 8438), secondary antibodies (Peroxidase‐conjugated Goat anti‐Rabbit IgG; ZSGB‐BIO, ZB‐2301, Beijing, China), Caspase‐3/7 Inhibitor I (ApexBio,A1925, Houston, TX, USA) and Pirarubicin (Selleck, Houston, TX, USA) were obtained commercially.

### 2.4 Gene silencing and transfection

Two different siRNA sequences targeted to different sites in MARCH1 mRNA were designed and provided by Genepharma (Shanghai, China). The sequences for the MARCH1 siRNA were as follows: for siRNA‐1, the sense sequence was 5′‐CAGGAGGUCUUGUCUUCAUTT‐3′, and the antisense sequence was 5′‐AUGAAGACAAGACCUCCUGTT‐3′; for siRNA‐2, the sense sequence was 5′‐GGUAGUGCCUGUACCACAATT‐3′, and the antisense sequence was 5′‐UUGUGGUACAGGCACUACCTT‐3′. The negative control siRNA (non‐target siRNA) was also purchased from GenePharma; the sense sequence was 5′‐UUCUCCGAACGUGUCACGUTT‐3′, and the antisense sequence was 5′‐ACGUGACACGUUCGGAGAATT‐3′. The cells were seeded (3 × 10^5^ per well) on 6‐well culture dishes to 30%‐50% confluence and transfected with 60 nmol/L siRNA‐MARCH1 using 6 μL lipofectamine 2000 (Thermo Fisher, Waltham, MA, USA) in accordance with the manufacturer's instructions. The plasmid of MARCH1 overexpression was also designed and provided by Genepharma (Shanghai, China). The cells were transfected with the MARCH1 plasmid and then treated with G418 for 2 weeks. Finally, Western blotting was used to test the knockdown or overexpression efficiency.

### 2.5 Western blot analysis

The lysates were boiled in SDS polyacrylamide gel electrophoresis (SDS‐PAGE) sample loading buffer for 5‐10 minutes at 99°C and run on SDS‐PAGE with 12% gels. The gels were transferred to polyvinylidene difluoride (PVDF) membrane (Solarbio, Beijing, China), and after blocking in 5% nonfat milk in a mixture of tris‐buffered saline and Tween 20 (TBST) with gentle agitation for 2–3 h, the membrane was left overnight with the primary antibody at 4°C and then incubated with the secondary antibody for 2 hours at room temperature. The enhanced chemiluminescence (ECL) reaction was performed with a super ECL kit (Novland, Shanghai, China). Then, the membranes were imaged, and images were analysed by using ImageJ. All the experiments were performed in triplicate.

### 2.6 Cell proliferation assay

The cell proliferation assay was performed according to the manufacturer's instructions for the Cell Counting Kit‐8 (CCK‐8) assay (Biosharp, Beijing, China). A total of 5 × 10^3^ cells were seeded in 96‐well plates and then treated according to the experimental requirements. CCK‐8 reagent was added to each well, and after incubation with the reagent for 1 hour at 37°C. The absorbance at 450 nm was measured using a spectrophotometer (SpectraMax M2, Shanghai, China). All the experiments were performed in triplicate.

The colony formation assay was performed as described previously.^14^ Briefly, the cells were seeded in 6‐well plates at a density of 5 × 10^3^ cells per well, transfected with siRNA after 24 hours of incubation at 37°C and 5% CO_2_ in a humidified incubator, and grown for over 12 days. The colonies were stained with 0.1% crystal violet solution (Solarbio, Beijing, China), washed with water, dried, imaged and counted according to colony number. But, the cells transfected with the MARCH1 plasmid were kept in culture with G418 for 2 weeks and then counted the numbers of the colony. Also, the cells treated with THP were washed with phosphate buffered saline (PBS) after 6 hours in 6‐well plates and then cultured as described previously.^15^ All the experiments were performed in triplicate.

### 2.7 Cell apoptosis assay

The apoptosis assays were performed using an annexin V‐FITC and propidium iodide (PI) apoptosis detection kit (KeyGEN Biotech, Nangjing, China). Briefly, the cells were stained with annexin V‐FITC and PI according to the manufacturer's instructions. Fluorescence signals from at least 10 000 cells were evaluated using a flow cytometer, and the apoptosis rate of the samples was immediately determined by flow cytometry. All the experiments were performed in triplicate.

### 2.8 Wound healing assay

The wound healing assay process was described previously.^15^ Briefly, the cells were cultured to 90% confluence, wounded by 200 μL pipette tip, washed with PBS and supplemented with a fresh medium with 1% FBS containing 2 μg/mL mitomycin C. The cell migration into the wounded area was photographed at different time points using 10 × objectives in an Olympus photomicroscope. The wound healing migration area was measured and analyzed using an Image‐Pro plus 6.0. All the experiments were performed in triplicate.

### 2.9 Transwell migration and invasion assay

Cellular migration and invasion assay were performed using 6.5 mm transwell insert chambers. Briefly, the cells (2.0 × 10^5^) were cultured in medium with 1% FBS, placed in the upper chamber with a 8.0 μm pore polycarbonate membrance (Corning, Kennebunk, ME, USA), and covered with a Matrigel (Corning) for 2‐5 hours at 37°C before the cells were added. Then, the medium with 20% FBS was added to the down chamber. After 12‐24 hours in the culture, the upper chamber was washed with water, dried, fixed with 4% paraformaldehyde at room temperature for 20 minutes, and stained with 0.1% crystal violet for 20 minutes for visualization. However, there was no need for the Matrigel coating for the cellular migration assay. All the experiments were performed in triplicate.

### 2.10 Mice and treatment

Female BALB/C athymic nude mice, aged 4 weeks, were purchased from Vital River Laboratories (Beijing, China) and allowed 1 week of acclimatization to their new surroundings. Then, these mice were housed in temperature‐controlled rooms with a 12 hours alternating light–dark cycle in the Specific Pathogen Free animal laboratory. Previous studies have described HepG2 cells can form subcutaneous tumours in nude mice.^16^, ^17^ Briefly, HepG2 cells (1 × 10^7^) were injected subcutaneously into the dorsal region near the hind leg of the nude mice. When the tumour volumes reached approximately 200 mm^3^, 18 mice with equivalently sized tumours were randomized into three groups. The animals were treated with an intra‐tumoural, multi‐point injection every 3 days with 25 μL PBS (blank control group) or 20 μL PBS with complexes of 15 μg siRNA, a set of 2′‐o‐Me and 5′cholesterol‐modified MARCH1 siRNA (MARCH1 siRNA‐1 treatment group), or negative siRNA (non‐target siRNA treatment group) together with 5 μL lipofectamine 2000, as previous studies have described^.^ 18, 19 The tumours were measured twice a week, and the tumour volumes were calculated by using the following formula: volume = (A × B^2^)/2, where A is the larger and B is the smaller diameter. After 4 weeks, all the mice were killed after magnetic resonance imaging (MRI) was performed, and the tumours were collected for histological analysis. Serial sections of tumour tissues were stained with haematoxylin and eosin (H‐E), and immunohistochemistry (IHC) was performed. All the mice were maintained in the Specific Pathogen Free animal laboratory of Binzhou Medical University, and all the animal studies were performed according to protocols approved by the Animal Ethics Committee of Binzhou Medical University.

### 2.11 MRI

Small‐animal MRI was performed by using a high field 7.0 Tesla MRI system (Bruker BioSpec 70/20USR, Karlsruhe, Germany). The three groups of nude mice were respectively placed in an animal bed, which was equipped with circulating warm water to sequentially regulate body temperature and were anesthetized with 1%‐2% inhaled isoflurane (Ruiward Life Technology Co., Ltd., Shenzhen, China) during the MRI. T1‐weighted imaging (T1WI), T2‐weighted imaging (T2WI), and diffusion‐weighted imaging (DWI) of the nude mice were performed using a nonmagnetic stereotactic wrist coil with a cylindric surface coil with a 5.0‐cm internal diameter positioned directly over the xenograft tumour area using the following protocol. T1‐weighted images with a fast low angle shot (FLASH) and fat saturation were acquired first and were performed by using the following parameters: repetition time (TR), 194.87 ms; echo time (TE), 2.60 ms; flip angle, 40 degree; slices orientation, Axial; slice thickness, 1 mm; 15 slices; matrix, 320 × 320; and field of view, 40 × 40. The fast spin echo (FSE) T2‐weighted sequence with fat saturation was performed by using the following parameters: TR, 1986.57 ms; TE, 34.37 ms; echo spacing, 11.457 ms; orientation, Axial; section thickness, 1 mm; 15 slices; matrix, 512 × 512; and field of view, 40 × 40. A coronal T2WI sequence with fat saturation was performed by using the following parameters: TR, 1764.99 ms; TE, 27.00 ms; echo spacing, 9.00 ms; section thickness, 1 mm; 10 slices; matrix, 320 × 320; and field of view, 40 × 40. Thereafter, an axial respiratory‐triggered single‐shot echo diffusion‐weighted sequence with fat saturation was performed by using the following parameters: b values of 650 seconds/mm^2^; TR, 2500.00 ms; TE, 33.00 ms; slice thickness, 1 mm; 15 slices; matrix, 128 × 128; and field of view, 40 × 40. The total acquisition time was about 60 minutes.

### 2.12 Histopathology and Immunohistochemistry

After the euthanization of the mice, the xenografts were fixed in 4% paraformaldehyde, dehydrated and embedded into paraffin wax blocks. The embedded‐tissues were cut into 5‐μm thick sections placed on adhesion microscope slides (Citoglas, China) and stained with H‐E (Novland, China). The immunohistochemistry was performed using the antibody MARCH1 (Bioss, bs‐9335, dilution 1:800). H–E and IHC were done as previously described.^14^, ^17^ Briefly, immunostaining analysis was independently performed by two clinical pathologists. Five fields were randomly selected per sample. Staining intensity of tumour cells and non‐cancers cells was assessed. The intensity of staining was scored as follows: 0 (negative), 1 (weakly positive), 2 (moderately positive) or 3 (strongly positive).

### 2.13 Statistical analysis

The statistical analyses were conducted using SPSS 17.0 and GraphPad Prism 5.0 software. Two‐tailed Student's *t* tests were used to test the significance of the differences between the two groups. All the data are represented as mean ± SD. * *P *<* *0.05 and **P <* *0.01 were considered statistically significant.

## 3 RESULTS

### 3.1 MARCH1 is up‐regulated in HCC tissues and cell lines

**Figure 1:**
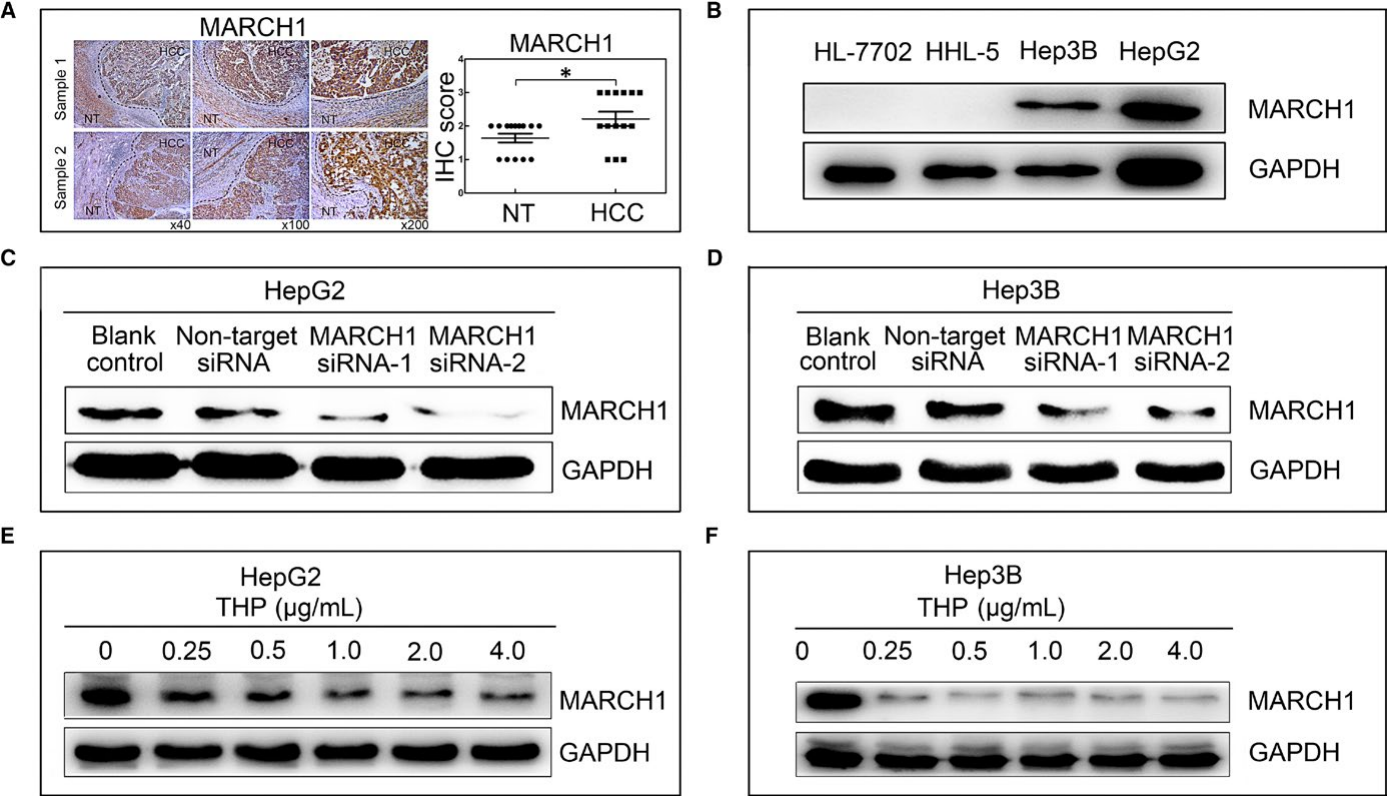
MARCH1 was highly expressed in the human hepatocellular carcinoma (HCC) tumour samples and cell lines (Hep3B and HepG2). A, Immunohistochemistry (IHC) analyses showing increased MARCH1 expression in liver tissue from patients with HCC compared with adjacent non‐tumour (NT) liver tissue; and the IHC score of MARCH1 in 14 cases. B, Western blotting assay showing the expression of MARCH1 in the four cell lines. C and D, Western blotting analysis was used to assay the interference efficiency of the two sequences of MARCH1 siRNA in the HepG2 and Hep3B cells for 48 h. E and F, Western blotting assay showed the MARCH1 protein levels in the HepG2 and Hep3B cells treated with pirarubicin (THP) for 24 h and 48 h in different concentrations, respectively. All the data in this figure are represented as mean ± SD. *P <* *0.05

To investigate the role of MARCH1 in HCC cells, here, we first detected the expression of MARCH1 in human liver samples, several human HCC cell lines and two normal human hepatocyte cell lines by immunohistochemical and western blot analyses, respectively. The MARCH1 level was highly expressed in six of 14 (45%) cases where HCC liver tissue was compared with the adjacent non‐cancerous liver tissues (Figure 1A). In addition, we further detected the levels of MARCH1 in the HCC cell lines (Hep3B and HepG2) and normal human hepatocyte cell lines (HL‐7702 and HHL‐5). The Western blot results showed that the MARCH1 protein was more elevated in the HCC cell lines than in the normal human hepatocyte cell lines (Figure 1B).

To further explore the biological function of MARCH1, we transiently depleted the MARCH1 expression in the HCC cells using two different effective sequences of siRNA interference (MARCH1 siRNA‐1 and MARCH1 siRNA‐2) and using the blank control (transfected negative siRNA) and non‐target siRNA (non‐transfected) groups as the negative controls (Figure 1C,D). Similarly, THP, an anthracycline anticancer drug, is clinically approved for treating various cancers and as a first‐line treatment chemotherapeutic for advanced HCC patients.^6^, ^20^ Interestingly, we found that THP could suppress MARCH1 expression in proteins. For this, we analysed MARCH1 protein levels by Western blot analysis in the HepG2 and Hep3B cells treated by THP in different concentrations (0, 0.25, 0.5, 1.0, 2.0, 4.0 μg/ml) for 24 hours and 48 hours, respectively. The results showed that the MARCH1 protein expression was significantly decreased in the two cell lines in a dose‐dependent manner (Figure 1E,F).

### 3.2 Down‐regulated MARCH1 expression inhibited HCC cell proliferation

**Figure 2:**
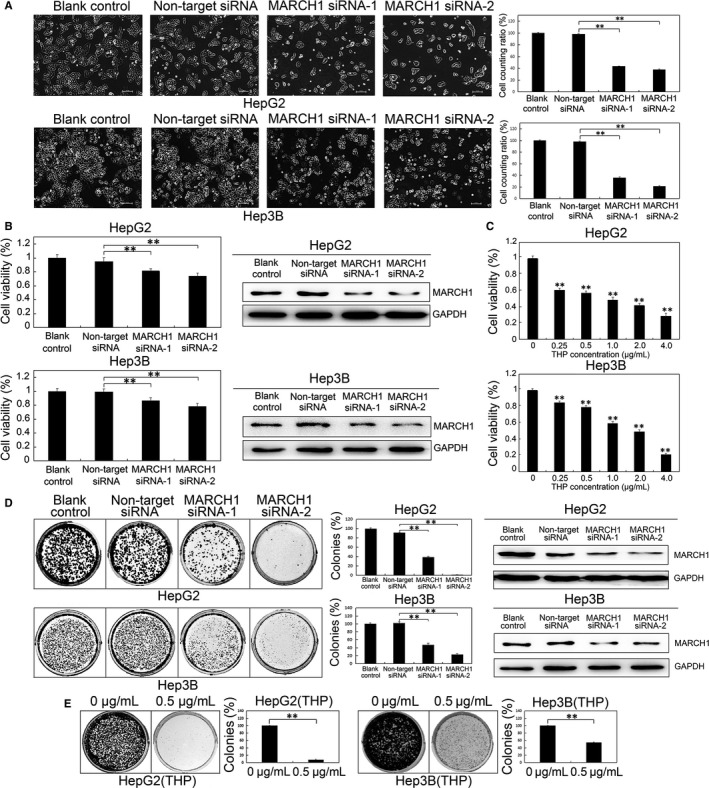
Down‐regulated MARCH1 inhibited human HCC cell proliferation. A, Representative microscope images of the HepG2 and Hep3B cells of MARCH1 siRNA interference for 48 h. B, The cell viability of the HepG2 and Hep3B cells transfected with MARCH1 siRNA (MARCH1 siRNA‐1 and MARCH1 siRNA‐2) and negative siRNA (non‐target siRNA) at 48 h post‐transfection is presented as a per cent of the cell viability attained by the non‐transfected cells (blank control). Western blotting assay was used to confirm the MARCH1 down‐regulation by siRNA. C, The cell viability of the HepG2 and Hep3B cells treated by THP in different concentrations for 24 h and 48 h, respectively, was assayed using a CCK‐8 cell proliferation assay,0 μg/mL was used as compared groups. D, Colony formation assay of transfected HepG2 and Hep3B cells. 5000 cells were seeded in 6‐well plates and grown for over 12 days. The colonies were stained with crystal violet solution, photographed and counted. The down‐regulation of the MARCH1 protein levels of colonies by siRNA for 6 d was confirmed by Western blot analysis. E, The colony formation assay of the HepG2 and Hep3B cells treated with THP. All the data in this figure are represented as mean ± SD. **P <* *0.01

After transfecting MARCH1 siRNA for 48 hours, the microscope images showed that the Hep3B and HepG2 cells treated by MARCH1 siRNA were significantly more impaired than those of the blank control and non‐target siRNA groups (*P *<* *0.01, *P *<* *0.01; *P *<* *0.01, *P *<* *0.01; Figure 2A). But, there was no significant difference in the level of the impairment of the cells between the blank control and non‐target siRNA groups. These results indicated that high MARCH1 expression in the HCC cells may promote the progression of the HCC cells.

To further determine whether MARCH1 had any effects on the HCC cells’ proliferation, we treated the HepG2 and Hep3B cells with targeted MARCH1 siRNAs and then conducted a CCK‐8 analysis of the cell viability. As shown in Figures 2B (*P *<* *0.01, *P *<* *0.01; *P *<* *0.01, *P *<* *0.01), The HepG2 and Hep3B cells transfected with targeted MARCH1 siRNAs decreased the cell viability compared with the cells transfected with negative siRNAs, which had no significant effect on cell growth, but there was no significant difference between the blank control and the non‐target siRNA group. Thus, the down‐regulation of MARCH1 by siRNA MARCH1 was confirmed.

Next, to determine if the MARCH1 down‐regulation by THP impacted the HCC cell growth, a CCK‐8 assay was conducted using cell viability. We found that THP inhibited the cell proliferation of the HepG2 and Hep3B cells in a dose‐dependent manner 0 μg/ml was used as compared groups (All *P *<* *0.01; Figure 2C). This demonstrated that the down‐regulation of the MARCH1 expression could be induced by both MARCH1 siRNA and THP; therefore, THP, as an inhibitor of MARCH1 and MARCH1 siRNA, may be used in the treatment of HCC in following studies.

We also tested the same effect of down‐regulating MARCH1 expression on cell proliferation in the HepG2 and Hep3B cells by using a colony formation assay. The number of colonies in the MARCH1 siRNA transfected cells was found to be reduced, and there was no significant difference in the number of colonies in the negative controls (*P *<* *0.01, *P *<* *0.01; *P *<* *0.01, *P *<* *0.01; Figure 2D); Western blotting assay was used to confirm the down‐regulation of MARCH1 of colonies for 12 days by siRNA. The same result that the number of colonies was reduced in the HepG2 and Hep3B cells treated with THP for 6 hours was verified (*P *<* *0.01; *P *<* *0.01; Figure 2E). These results indicated that the down expression of MARCH1 decreased the viability, the colony numbers and the size of the HCC cells. Furthermore, these data demonstrated that down‐regulated MARCH1 inhibited human HCC cell proliferation.

### 3.3 Down‐regulated MARCH1 expression promoted human HCC cell apoptosis

**Figure 3:**
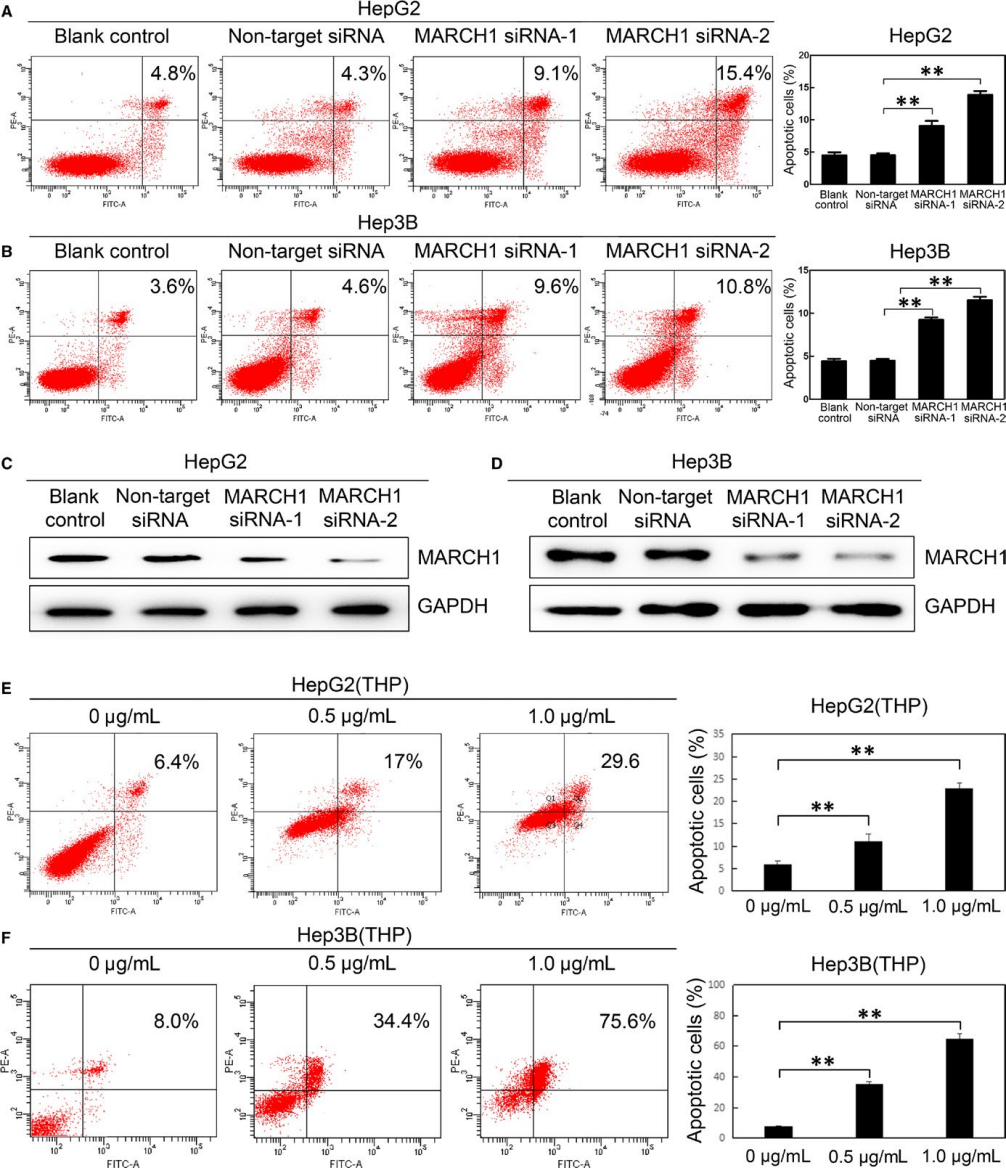
Down‐regulated MARCH1 induced human HCC cell apoptosis. A and B, The cell apoptosis ratio of the HepG2 and Hep3B cells transfected with the two sets of MARCH1 siRNA, negative siRNA and non‐transfected for 48 h, respectively. C and D, Western blotting assay was used to confirm the MARCH1 down‐regulation by siRNA. E and F, Cell apoptosis ratio of the HepG2 and Hep3B cells treated with THP in different concentrations for 24 h and 48 h, respectively. All the data in this figure are represented as mean ± SD. ** *P *<* *0.01

To verify whether MARCH1 knockdow also induced cell apoptosis, annexin V and propidium iodine staining followed by flow cytometric analysis was used to analyse cell apoptosis. The degrees of cell apoptosis in the HepG2 and Hep3B cells transfected with MARCH1 siRNA were higher than those in the cells transfected with negative siRNA (*P *<* *0.01, *P *<* *0.01; *P *<* *0.01, *P *<* *0.01; Figure 3A,B), and there was no significant difference in the degrees of cell apoptosis between the control and the non‐target siRNA groups. The MARCH1 knockdown by siRNA was confirmed by western blot analysis (Figure 3C,D). Additionally, we found that THP significantly promoted the apoptosis of both the HepG2 and Hep3B cells in a dose‐dependent manner, which might partially through the down‐regulation of the MARCH1 expression (*P *<* *0.01, *P *<* *0.01; *P *<* *0.01, *P *<* *0.01; Figure 3E,F). These results indicated that the decrease in cell proliferation, seen upon the suppression of the MARCH1 expression, was not only due to the inhibition of the cell proliferation, but also the enhanced cell apoptosis.

### 3.4 MARCH1 knockdown impaired human HCC cell migration and invasion

**Figure 4:**
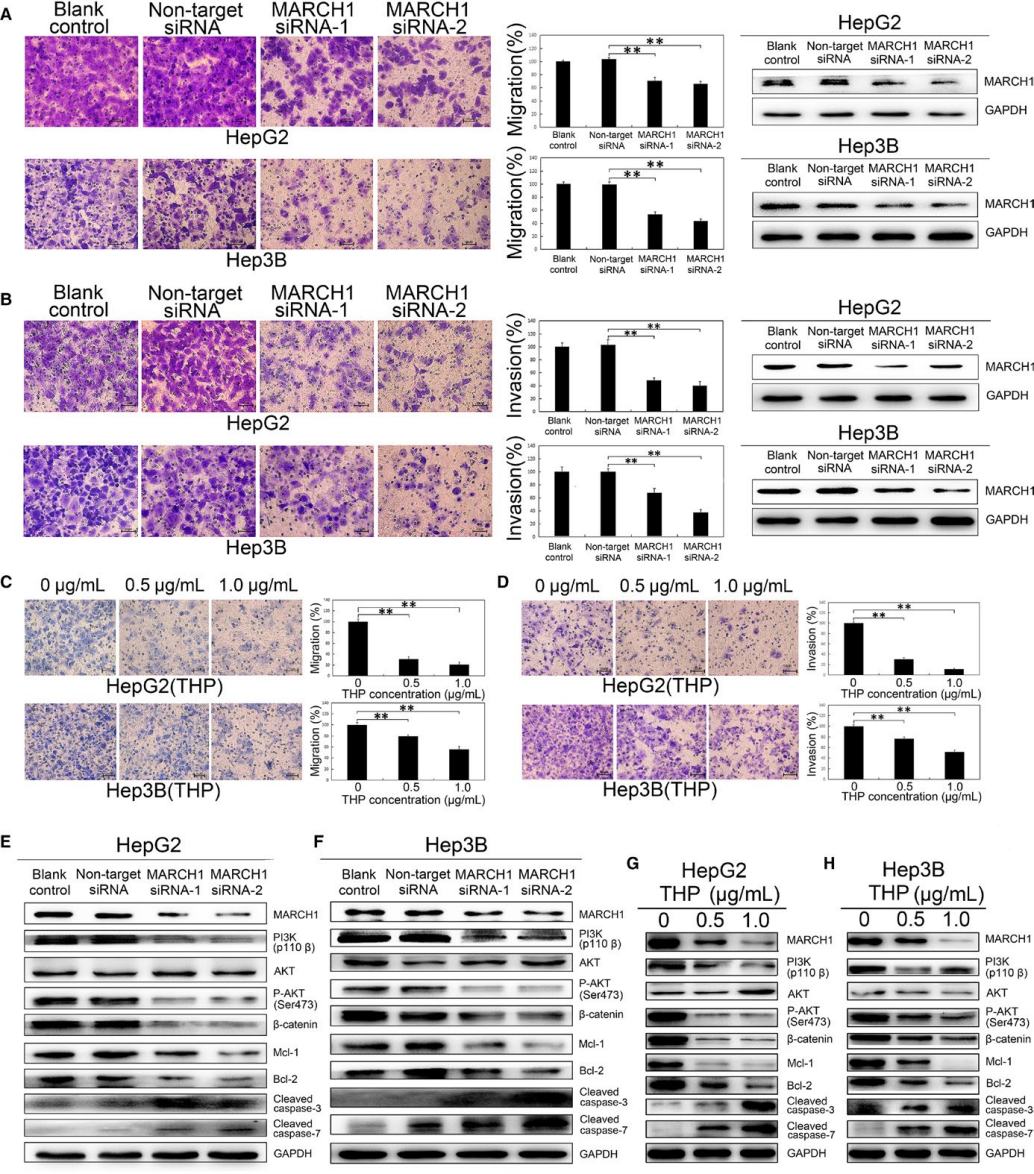
MARCH1 knockdown inhibited human HCC cell migration and invasion through down‐regulated PI3K/P‐AKT/β‐catenin pathways and induced apoptosis. A and B, In vitro migration and invasion assay for the negative control and MARCH1 siRNA in the HepG2 and Hep3B cells. Knockdown of the MARCH1 protein with siRNAs in the human HepG2 and Hep3B HCC cells. C and D, In vitro migration and invasion assay for the HepG2 and Hep3B cells with THP in concentrations of 0, 0.5 and 1.0 μg/mL. E and F, Protein expression of MARCH1, PI3K, AKT, P‐AKT, β‐catenin, MCL‐1, BCL‐2, Cleaved caspase‐3 and Cleaved caspase‐7 in the HepG2 and Hep3B cells. G and H, And in the HepG2 and Hep3B cells with 0, 0.5 and 1.0 μg/mL THP concentrations. All the data in this figure are represented as mean ± SD. **P <* *0.01

To further investigate the effects of MARCH1 on the HCC cell migration and invasion, transwell assays were performed. The transwell migration assays revealed that the down‐regulation of MARCH1 led to a dramatic decrease in the cell motility of the HepG2 and Hep3B cells (*P *<* *0.01, *P *<* *0.01; *P *<* *0.01, *P *<* *0.01; Figure 4A), The MARCH1 knockdown was confirmed by Western blotting. Moreover, the matrigel invasion assays showed that with the MARCH1 knockdown, the HepG2 and Hep3B cells exhibited a marked reduction in cell invasion (*P *<* *0.01, *P *<* *0.01; *P *<* *0.01, *P *<* *0.01; Figure 4B). There was no significant difference in the cell migration and invasion ratio between the blank control and non‐target siRNA groups. The MARCH1 knockdown was effective. Similar results were obtained with THP in cell migration and invasion assays, showing that THP, as an inhibitor of MARCH1, significantly inhibited the cell migration (*P *<* *0.01, *P *<* *0.01; *P *<* *0.01, *P *<* *0.01; Figure 4C) and invasion (*P *<* *0.01, *P *<* *0.01; *P *<* *0.01, *P *<* *0.01; Figure 4D) in the HepG2 and Hep3B cells in a dose‐dependent manner as well. These results further suggested that MARCH1 silencing decreases HCC cell migration and invasion.

### 3.5 MARCH1 knockdown suppressed human HCC cell progression by down‐regulating PI3K‐AKT‐β‐catenin pathways

Recent studies have indicated that approximately 50% of hepatocellular carcinoma cases display aberrant PI3K‐AKT and Wnt‐β‐catenin signalling pathways, respectively.^21^ Meng et al. demonstrated that MARCH1 silencing by siRNA suppressed cell development and progression via the down‐regulation of the Wnt/β‐catenin pathway in ovarian cancer.^13^ To explain how down‐regulated MARCH1 inhibited HCC cell proliferation, migration and invasion and promoted cell apoptosis, we explored the underlying molecular changes downstream of the MARCH1 perturbation. Here, we addressed the molecular effects of the down‐regulated MARCH1 by using western blotting assay. The decreased expression of PI3K, AKT phosphorylation, β‐catenin, Mcl‐1 and Bcl‐2 and the increased expression of Cleaved caspase‐3 and Cleaved caspase‐7 were detected in the HepG2 and Hep3B cells transfected with MARCH1 interference (Figure 4E,F). Interestingly, consistent with the following finding that THP drastically induced the down‐regulation of MARCH1 and further significantly inhibited PI3K‐activated downstream targets, such as P‐AKT, β‐catenin, MCL‐1 and BCL‐2, THP up‐regulated the expression of Cleaved caspase‐3 and Cleaved caspase‐7 in a dose‐dependent manner in the HepG2 and Hep3B cells (Figure 4G,H). To further identify the specific molecular mechanisms of MARCH1 on human HCC cells, we used a caspase‐3/7 inhibitor to validate the caspase pathways. A flow cytometer showed the cell apoptosis ratio of the HepG2 and Hep3B cells with THP, and the caspase‐3/7 inhibitor was decreased (*P *<* *0.01, *P *<* *0.01; *P *<* *0.01, *P *<* *0.05; Figure S1A‐C). Thus, the results collectively suggested that MARCH1 silencing inhibited PI3K‐AKT‐β‐catenin axis activity.

### 3.6 MARCH1 overexpression promoted human HCC cell proliferation, migration and invasion by up‐regulating PI3K‐AKT‐β‐catenin pathways

**Figure 5:**
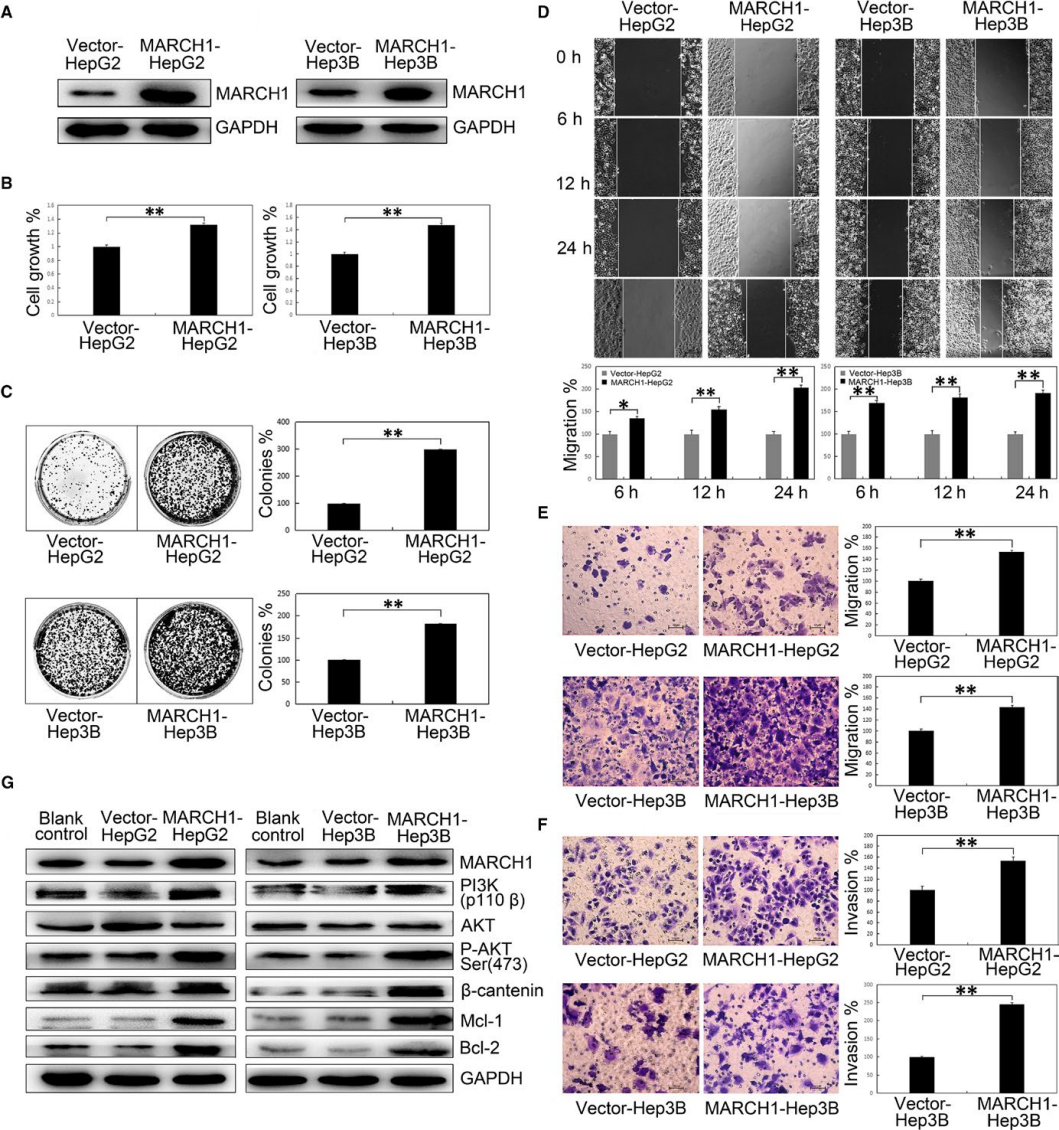
MARCH1 overexpression accelerated human HCC progression of proliferation, colony formation, wound healing, transwell migration and invasion by activating the PI3K/P‐AKT/β‐catenin pathways. A, Overexpression of the MARCH1 protein with empty vectors and plasmids in the human HepG2 and Hep3B HCC cells. In vitro (B) cell proliferation and (C) colony formation in the HepG2 and Hep3B cells with empty vectors and MARCH1 overexpression. D, In vitro wound healing assay in the HepG2 and Hep3B cells with empty vectors and MARCH1 overexpression. 40 × images show the wound size at 0, 6, 12 and 48 h after the scratch. E and F, In vitro transwell migration and invasion in the HepG2 and Hep3B cells with empty vectors and MARCH1 overexpression. G, Protein expression of MARCH1, PI3K, AKT, P‐AKT, β‐catenin, MCL‐1, BCL‐2 in the human HepG2 and Hep3B cells transfected with empty vectors and plasmids. All the data in this figure are represented as mean ± SD. *P < 0.05, **P <* *0.01

Next, to fully validate the biological function of MARCH1, we overexpressed MARCH1 in the HCC cell lines. MARCH1 and an empty vector plasmid were transfected into the HCC cells using lipofectamine 2000. The transfected cells were screened for more than 2 weeks in incubation with G418. Stable single clones of the HepG2 and Hep3B cells were selected, harvested and tested with Western blotting analysis for transfected efficiency (Figure 5A). We confirmed that the overexpression of MARCH1 significantly increased the HepG2 and Hep3B cell viability capability (*P *<* *0.01, *P *<* *0.01; Figure 5B), increased the number of colonies in the HCC cells (*P *<* *0.01, *P *<* *0.01; Figure 5C), and significantly accelerated the cell migration at 0, 12 and 24 hours after the scratch in the cell wound healing assay, respectively (*P *<* *0.05, *P *<* *0.01, *P *<* *0.01; *P *<* *0.01, *P *<* *0.01, *P *<* *0.01; Figure 5D). Similar results were obtained in the cell transwell migration and invasion assays. The MARCH1 up‐regulation of the HepG2 and Hep3B cells also significantly accelerated the cell migration and invasion compared with the empty vector transduced cells (*P *<* *0.01, *P *<* *0.01, *P *<* *0.01, *P *<* *0.01; Figure 5E,F).

Additionally, to identify the molecular mechanisms of the MARCH1 overexpression in the accelerated HCC aggressiveness associated with the activation of downstream molecules of PI3K‐AKT‐β‐catenin, Western blot analysis was used to detect the relevant cell function of the regulatory molecules. Notably, pI3K, phosphorylation of AKT and β‐catenin were elevated in the MARCH1 overexpression in both the HepG2 and Hep3B cells. In addition, the levels of downstream Mcl‐1 and Bcl‐2 were also increased after MARCH1 overexpression (Figure 5G). Therefore, these findings support the possibility that MARCH1 plays a promotional role in HCC development and aggressiveness by activating the PI3K‐AKT‐β‐catenin pathways.

### 3.7 MARCH1 silencing inhibited tumour growth in nude mice via down‐regulating of PI3K‐AKT‐β‐catenin pathways

**Figure 6:**
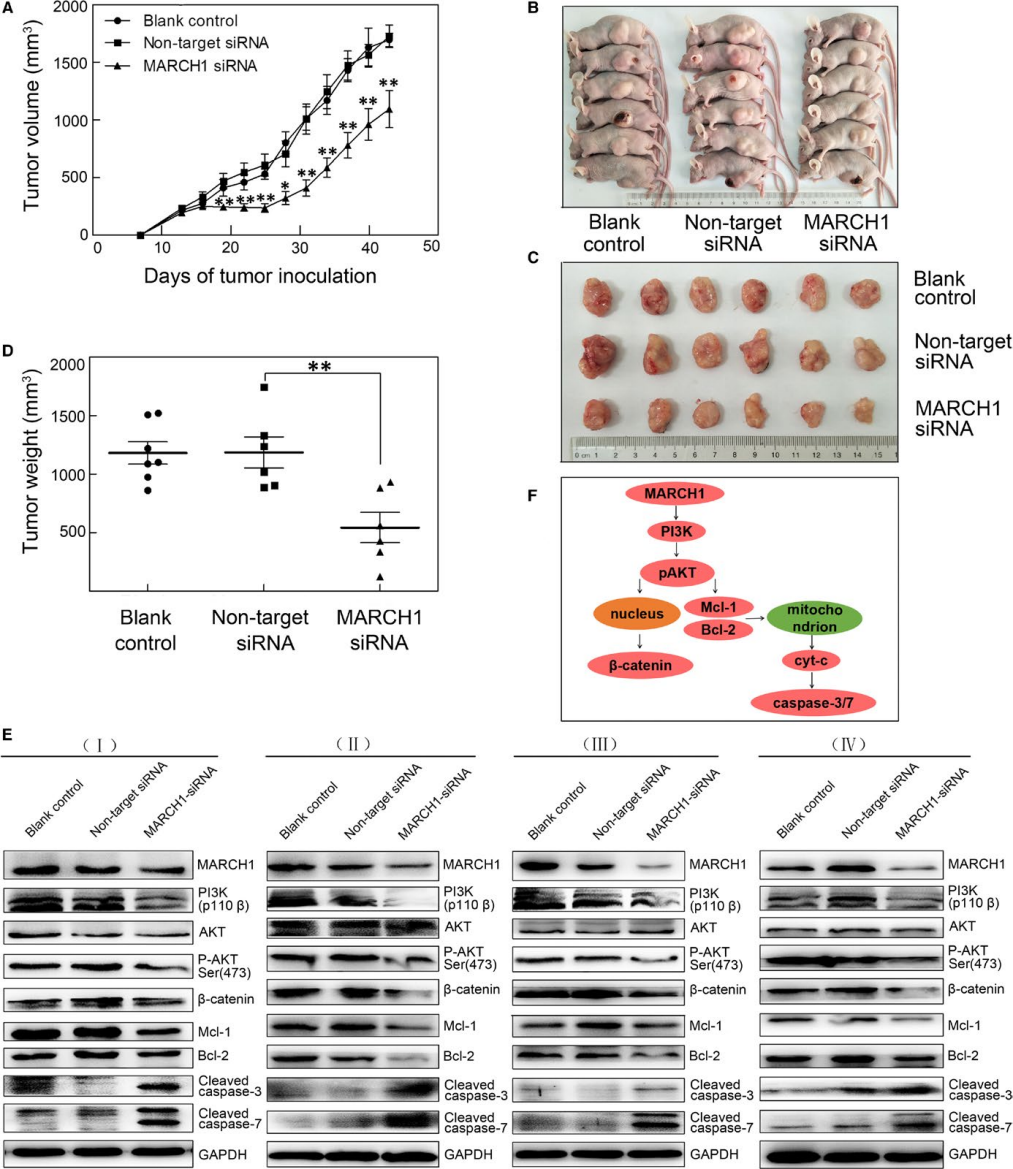
MARCH1 silencing inhibited tumour growth in nude mice via the down‐regulating of the PI3K/P‐AKT/β‐catenin pathways. A, Tumour growth curves for different therapy groups of PBS‐injected, negative siRNA‐injected and MARCH1‐injected tumours, respectively. B and C, Images of representative mice for different therapy groups. D, Tumour weight for different therapy groups. E, Protein expression of MARCH1, PI3K, AKT, P‐AKT, β‐catenin, MCL‐1, BCL‐2, Cleaved caspase‐3 and Cleaved caspase‐7 in the three different therapy tumour tissues with PBS‐injected, negative siRNA‐injected and MARCH1‐injected for four groups samples. F, Model for MARCH1 in PI3K/AKT/β‐catenin signalling. MARCH1 induces PI3K membrane recruitment, which activates phosphorylation and recruitment of AKT, leading to the promotion of β‐catenin expression. Subsequently, the phosphorylation and degradation of β‐catenin is decreased. AKT activation can trigger Mcl‐1 and Bcl‐2 up‐regulation, thus blocking the cytc/caspase‐3/7 pathway. All the data in this figure are represented as mean ± SD. *P < 0.05, **P <* *0.01

Next, we aimed to validate the role MARCH1 plays in HCC tumour growth in vivo. The effect of MARCH1 silencing on tumour growth in vivo was examined using nude mouse subcutaneous xenograft models. MARCH1 siRNA, negative siRNA and PBS were injected into nude mice subcutaneous tumours, respectively, at multiple points. After 4 weeks, the body weight of the nude mice showed no obvious change (data not shown). The average tumour volume and weight of the MARCH1 siRNA group (MARCH1 siRNA‐injected) were markedly reduced compared with the non‐target group (negative siRNA‐injected) (*P *<* *0.01; Figure 6A‐D), while there were no differences in the tumour volume and weight between the blank control (PBS‐injected) and non‐target siRNA groups. These results conversely indicated that MARCH1 could significantly promote tumour growth and the progression of HCC.

Furthermore, to further investigate how MARCH1 silencing impairs tumour progression, we performed a Western blotting analysis to determine the expression of the PI3K‐related pathway markers in the three groups’ tumours with different treatments. The Western blotting showed a down‐regulated expression of MARCH1, PI3K, P‐AKT, β‐catenin, Bcl‐2 and Mcl‐1 and an up‐regulated expression of pro‐apoptosis‐related Cleaved caspase‐3 and Cleaved caspase‐7 in the HCC tumours injected with MARCH1 siRNA compared with the negative controls (Figure 6E). The results conversely showed that the tumours expressing high MARCH1 tended to have up‐regulation of the PI3K‐AKT‐β‐catenin pathways, collectively, revealing the strong pro‐tumorigenic ability of MARCH1.

**Figure 7:**
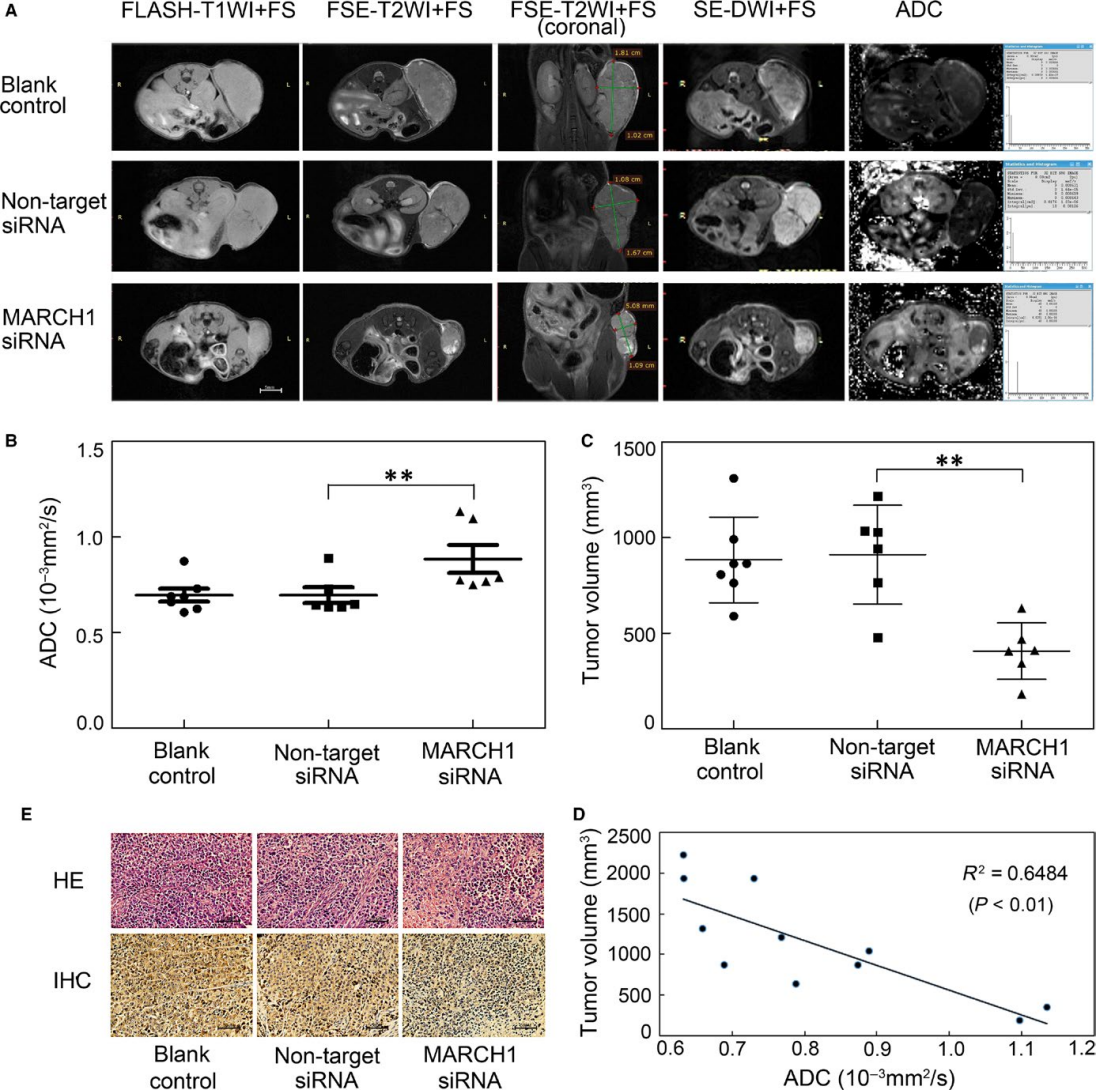
Magnetic resonance imaging (MRI), hematoxylin and eosin (H–E) and IHC of human HCC tumours. A, T1‐weighted MRIs, axial and coronal T2‐weighted MRIs, diffusion‐weighted MRIs and apparent diffusion coefficient (ADC) maps of the PBS‐treated, negative siRNA‐treated and MARCH1 siRNA‐treated tumours. B, Average tumour ADC in the PBS‐treated, negative siRNA‐treated, and MARCH1 siRNA‐treated tumours. C, Average tumour volume acquired on the coronal T2WI in the PBS‐treated, negative siRNA‐treated and MARCH1 siRNA‐treated tumours. D, Positive correlation between the ADC value and tumour volume. E, H–E histology and MARCH1 IHC in the PBS‐treated, negative siRNA‐treated and MARCH1 siRNA‐treated tumour tissue. All the data in this figure are represented as mean ± SD. **P <* *0.01

In addition, DW‐MRI is a functional imaging technique that can evaluate the water diffusion process in vivo and is sensitive to microstructural changes occurring at the cellular level. The apparent diffusion coefficient (ADC), a quantitative parameter of DW‐MRI, has been found to enable the assessment of tumour cellularity, necrosis, cell apoptosis and cell density, which is often used as a useful non‐invasive biomarker for the early detection of treatment response.^22^, ^23^, ^24^ Therefore, MRI was firstly used to elucidate the responses of the tumours to the treatment of MARCH1 siRNA in our study. The representative T1‐weighted MRIs, axial and coronal T2‐weighted MRIs, diffusion‐weighted MRIs and ADC maps of the PBS‐treated, negative siRNA‐treated and MARCH1 siRNA‐treated tumours were clearly exhibited (Figure 7A). We found that the ADC measured by DW‐MRI in the MARCH1 siRNA‐treated tumours was significantly higher than that in the negative siRNA‐treated tumours (*P *<* *0.01; Figure 7B), with no significant difference between the control PBS‐treated and negative siRNA‐treated tumours. A similar result showed that the MARCH1 siRNA‐treated tumours’ volume, acquired by the coronal T2‐weighted MRIs, was significantly decreased (*P *<* *0.01; Figure 7C). The negative correlation between the ADC value and tumour volume was validated (*P *<* *0.01; Figure 7D). Additionally, H–E staining in the MARCH1 siRNA‐treated tumours showed more tumour necrosis and loose cell spacing than did the negative siRNA‐treated and PBS‐treated tumours. The lowest level of MARCH1 staining in the MARCH1 siRNA‐treated tumours was validated (Figure 7E). Thus, these results further indicated that MARCH1 silencing induced tumour apoptosis and inhibited tumour growth.

## 4 DISCUSSION

Liver cancer is a complex, multifactorial and multistep process; aberrant expression of tumour suppressor genes and oncogenes or abnormal protein alterations is involved in the initiation and progression of cancer.^3^ Due to the difficulty of early‐stage HCC diagnosis and its low survival rate,^4^ the dysfunction of multiple metabolic pathways result in a neoplastic phenotype. Therefore, a better understanding of the aberrant expression of genes and specific metabolic genes in HCC would contribute to the design of novel therapeutic strategies. In this study, we first demonstrated that MARCH1 was aberrantly and highly expressed in HCC samples and cells lines and that the knockdown expression of MARCH1 led to a dramatic decrease in the proliferation, migration, invasion and increase in apoptosis via the regulating of PI3K and its downstream AKT‐β‐catenin pathway. By contrast, MARCH1 overexpression had the opposite effect. Here, we first report that the PI3K‐Akt‐β‐catenin signalling pathway, as a target of MARCH1, may help us to better understand how MARCH1 drives tumour development and progression and may help pave the way for the design of novel therapeutic protocols in human HCC treatment.

Membrane‐associated RING‐CH‐1 is a member of the MARCH family of membrane‐associated E3 ubiquitin ligase, which can ubiquitinate and down‐regulate the surface expression of some immune‐associated membrane proteins. Specifically, MARCH1 was shown to be capable of ubiquitinating MHCII and mediating intracellular localization and lysosomal degradation of MHC II in DCs and B cells.^25^, ^26^, ^27^, ^28^, ^29^ Ubiquitin has an ability to control membrane protein expression and localization and exert significant influence on cellular function. In the immune system, ubiquitination plays an important role in cellular function regulation.^30^, ^31^ As an E3 ubiquitin ligase, MARCH1 can ubiquitinate various substrates. Previous studies have reported that MARCH1 ubiquitinates cell surface insulin receptor levels to regulate insulin sensitivity 32 and ubiquitinates CD98 limits cell proliferation and clonal expansion.^33^ Recently, a study demonstrated that the adoptive transfer of MARCH1‐silenced autophagy‐deficient monocytic MDSCs (M‐MDSCs) significantly inhibited melanoma growth and induced a powerful anti‐tumour immune response in melanoma‐bearing mice.^34^ In ovarian cancer, studies have shown that the abnormal, high expression of MARCH1 seriously promoted tumour progression.^13^ This study suggested that MARCH1 regulates cellular processes, not only in immunity, but also in cell signal transduction. Here, in this study, we first found that MARCH1 is highly expressed in both human HCC samples and HCC cell lines, suggesting that MARCH1 plays an important role in HCC. Our results further demonstrated that the knockdown of MARCH1 obviously impaired the proliferation, migration, invasion and accelerated apoptosis of HCC cells through the inhibiting of PI3K‐AKT‐β‐catenin signalling in vivo and in vitro. Despite these important observations, the significance of MARCH1 in human cancer has not been fully investigated. Pirarubicin (THP) is an important chemotherapy agent of TACE that is usually used in HCC combination therapy.^6^, ^35^ Interestingly, we found that THP, as an inhibitor for MARCH1, could accelerate cell apoptosis and decrease the proliferation, migration and invasion in HCC cells also via the PI3K‐AKT‐β‐catenin signalling pathway. This result suggested that the powerful anticancer molecular mechanism of THP was partially mediated by the down‐regulated expression of MARCH1 in HCC cells. Although THP could down‐regulate the level of MARCH1 in protein but not in transcriptional level (data not shown), so the mechanism needs to be further. In all, the anticancer molecular mechanism of THP was partially targeting MARCH1 in HCC cells. More, MARCH1 overexpression was confirmed by transfection with plasmids on HCC cells. Our data showed that MARCH1 overexpression significantly accelerated human HCC cell proliferation, migration and invasion. Importantly, the MARCH1 knockdown caused by the intra‐tumour injection of siRNA targeting MARCH1 drastically reduced tumour growth in vivo in xenograft mouse models with human HCC. In this regard, here, we demonstrated that MARCH1 deletion does affect the biomarker of HCC tumours. The ADC value, as a non‐invasive biomarker and a sensitive biomarker to predict early treatment responses in various malignant tumours derived from DW‐MRIs, was used to assess tumour cellularity, necrosis, cell apoptosis and cell density.^22^, ^23^, ^24^, ^36^ Previous studies have shown an ADC increase following targeted agent treatment with cisplatin in an ovarian cancer model,^37^ sorafenib in breast cancer xenografts 38 and irinotecan in colon carcinoma xenografts.^39^ Hence, we used DW‐MRI to monitor the therapy response of the HCC xenografts under different therapy conditions. We found that the ADC values increased in the HCC tumours with the injection of MARCH1 siRNA. The ADC value increase was related to tumour necrosis and apoptotic cell death supported by H‐E, Western blotting assay, and T2‐weighted MRIs. H‐E staining showed more tumour necrosis, and the cell spaces became looser; Western blotting analysis detected high levels of cleaved caspase‐3 and cleaved caspase‐7; and T2‐weighted MRIs showed more high signal necrotic areas on the HCC tumours after MARCH1 siRNA therapy. Our data are consistent with earlier studies demonstrating that tumour ADC increase was associated with the induction of necrotic and apoptotic cell death.^39^ In addition, a previous study reported that ADC values of differentiated pancreatic ductal adenocarcinoma (PDAC) were higher than those of non‐differentiated PDAC xenografts.^40^ Therefore, our findings depicted that MARCH1 plays a promotional role in the development and progression of HCC in vitro and in vivo.

PI3K‐AKT‐mTOR and Wnt‐β‐catenin are known as important typical signalling pathways that promote cell proliferation, migration and invasion and inhibit cell apoptosis during stresses in HCC. Approximately 50% of hepatocellular carcinoma cases show aberrant, highly activated PI3K‐AKT‐mTOR and Wnt‐β‐catenin signaling.^21^ In multiple types of human cancer, the Type I insulin‐like growth factor receptor (IGF‐IR) and Insulin receptor (InsR) were activated when they received signals from extracellular growth factors or hormones, leading to membrane recruitment and the activation of PI3K, whereas PI3K activation directly or indirectly induced AKT phosphorylation, thereby promoting the activation of mTOR and the regulating of cell growth and metabolism.^41^, ^42^ In addition, the PI3K‐Akt‐β‐catenin signalling pathway is highly activated in many cancers, and AKT phosphorylation may directly mediate β‐catenin nuclear accumulation and transcriptional activation, promoting tumour development and progression.^43^, ^44^ Our observations showed that the MARCH1 knockdown by siRNA targeting MARCH1 or THP obviously suppressed PI3K activity and AKT phosphorylation and indirectly inhibited β‐catenin expression both in vitro and in vivo HCC. Conversely, we also showed that MARCH1 overexpression significantly activated PI3K, phosphorylated AKT, and accelerated β‐catenin translocation (Figure 6F). Our result was consistent with a study that showed that the MARCH1 knockdown results in NF‐κB and β‐catenin inhibition in ovarian cancer.^13^ Previous studies have demonstrated that MARCH1 was a negative regulator of INSR signalling in various cell types, including hepatocytes and white adipocytes. MARCH1 regulated INSRβ Lys^1079^, which is a potential substrate to regulate surface INSRb expression, and MARCH1 expression was regulated through a canonical FOXO1‐mediated mechanism.^32^ Recent studies have reported that anti‐apoptotic Mcl‐1 and Bcl‐2 were often overexpressed and played pivotal roles in malignancies, including in HCC. The expression of Mcl‐1 and Bcl‐2 can be regulated by Akt activation, thus regulating the cytc/caspase‐9/caspase‐3 pathway.^45^, ^46^, ^47^, ^48^ In this study, we found that the MARCH1 knockdown induced AKT inactivation and triggered Mcl‐1 and Bcl‐2 down‐regulation, thus activating cytc/caspase‐3/7 cascades, whereas MARCH1 overexpression activated AKT phosphorylation and failed to down‐regulate Mcl‐1 and Bcl‐2 efficiently, thus inhibiting the cytc/caspase‐3/7 axis (Figure 6F). Thus, this forcefully suggests that MARCH1 mediated HCC development and progression by regulating the PI3K‐AKT‐β‐catenin pathways and cytc/caspase‐3/7 cascades. Notably, our study also has some limitations; whether MARCH1 directly regulated IGF‐IR or InsR and whether the level of MARCH1 expression was mediated by the nuclear transcription factor FOXO1 or NF‐κB in HCC is unknown. The molecular mechanisms underlying the integration of multiple signalling elements remain obscure. Furthermore, the underlying functional impact and mechanism of MARCH1 require further study and future exploration in HCC patients.

## 5 CONCLUSIONS

In conclusion, our results demonstrate, for the first time, the potential role of MARCH1 in stimulating tumours in HCC. Moreover, MARCH1 could regulate the PI3K‐AKT‐β‐catenin signalling pathway in vitro and vivo, which is a crucial tumour‐related signalling axis in HCC. Although more in‐depth molecular mechanisms and tumorigenic effects for MARCH1 in HCC need to be further identified in the future, our findings have broad significance for the understanding of MARCH1′s behaviour and functions and provide a preliminary basis to explore MARCH1 as novel potential molecular therapeutic target for the development and progression of HCC treatment in the future.

## CONFLICTS OF INTEREST

The authors declare that they have no competing financial interests.

## AUTHOR CONTRIBUTION

Conceptualization, XH, MZ, ML and LX; Methodology, XL and ML; Formal Analysis, LX, HD and MZ; Investigation, XL, HD and ML; Resources, WY, XW, WL and GY; Writing – Original Draft Preparation, LX and ML; Writing – Review & Editing, XH and MZ; Funding Acquisition, ML and PW.

## Supporting information


** **

** **
