# Supplementary figures and images for: MARCH1 encourages tumour progression of hepatocellular carcinoma via regulation of PI3K‐AKT‐β‐catenin pathways
Source: J Cell Mol Med. 2019 Feb 22;23(5):3386–401. doi: 10.1111/jcmm.14235 (PMC6484336; doi:10.1111/jcmm.14235)

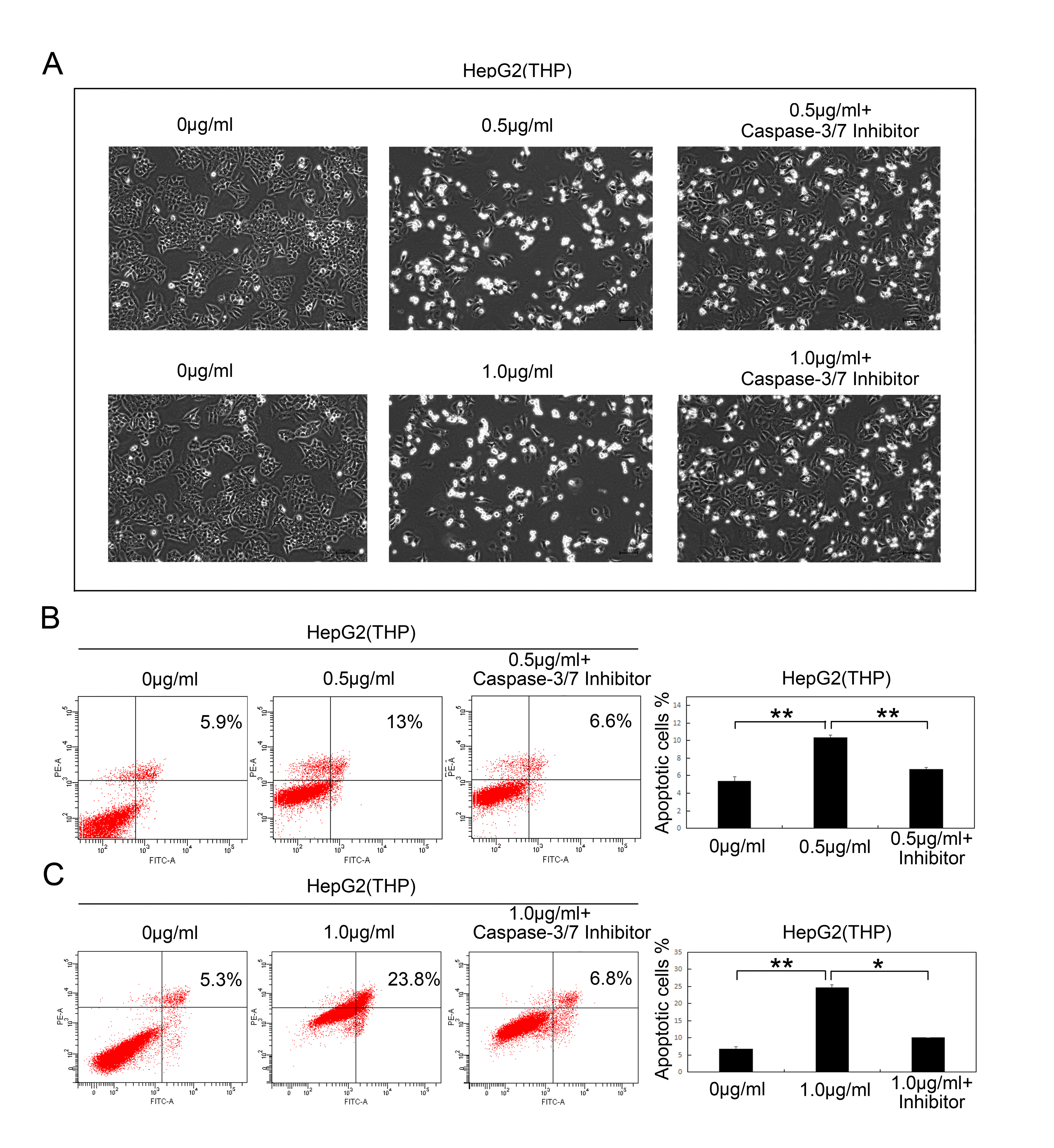

Supplement: Supplementary file 1 [file JCMM-23-3386-s001.tif]
