# Supplementary material for: MARCH1 encourages tumour progression of hepatocellular carcinoma via regulation of PI3K‐AKT‐β‐catenin pathways
Source: J Cell Mol Med. 2019 Feb 22;23(5):3386–401. doi: 10.1111/jcmm.14235 (PMC6484336; doi:10.1111/jcmm.14235)
Supplement: Supplementary file 2 [file JCMM-23-3386-s002.doc]

**
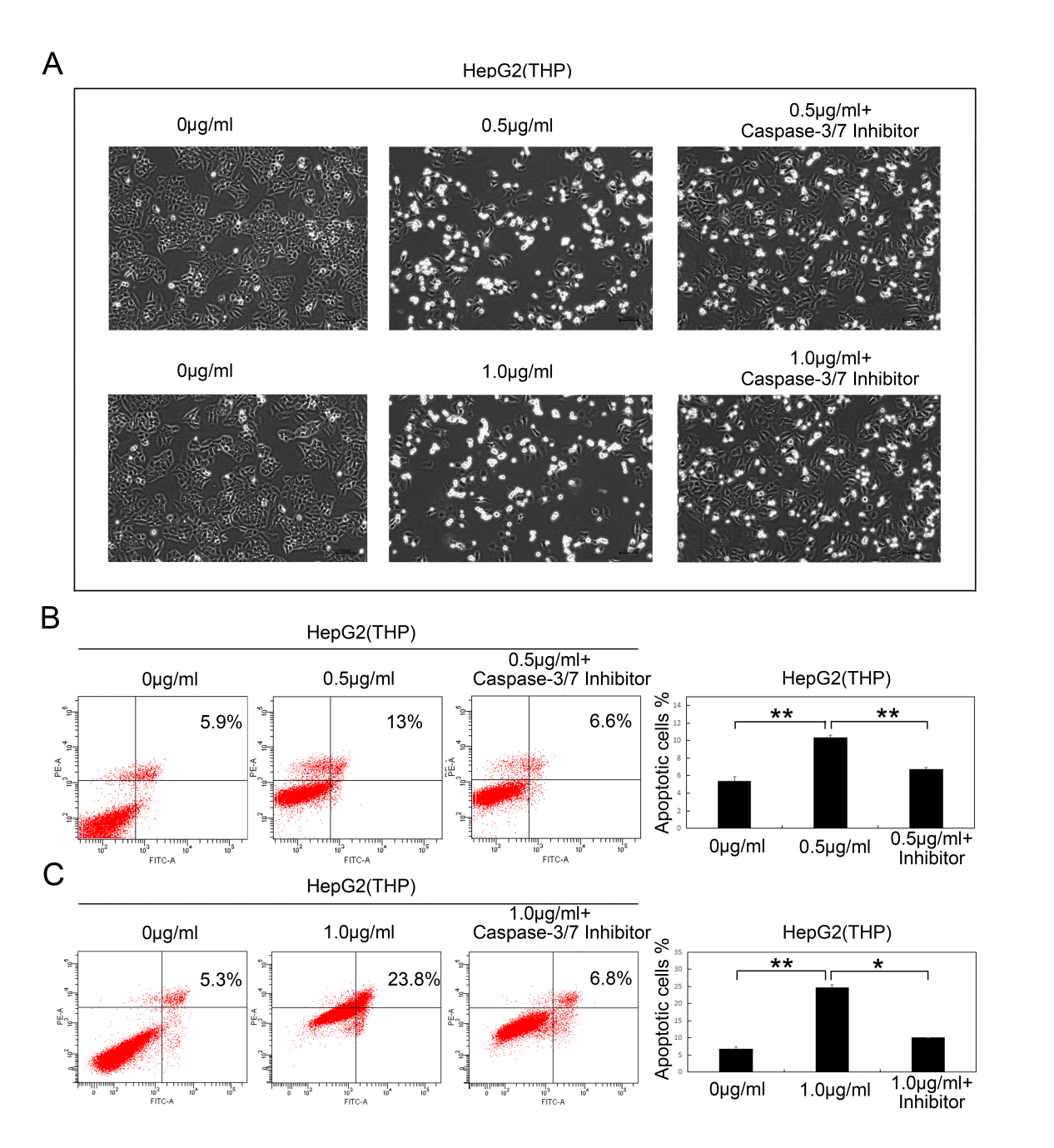
**

**Figure S1:** Caspase-3/7 inhibitor suppressed HCC apoptosis induced by pirarubicin (THP). (**A**) Images of human HepG2 and Hep3B cells with 0, 0.5, 1.0 μg/ml THP and plus caspase-3/7 inhibitor. (**B**,**C**) Flow cytometer showing the cell apoptosis ratio of HepG2 and Hep3B with THP and plus caspase-3/7 inhibitor.
